# Osmotic Demyelination Syndrome in the Setting of Normonatremia: A Case Report and Review of the Literature

**DOI:** 10.1155/crnm/6626539

**Published:** 2024-11-27

**Authors:** Rose V. Zach, Jeffrey F. Barletta, Victor Zach

**Affiliations:** ^1^University of Arizona College of Medicine, Phoenix, Arizona, USA; ^2^Department of Pharmacy Practice, College of Pharmacy, Midwestern University, Glendale Campus, Glendale, Arizona, USA; ^3^Arizona College of Osteopathic Medicine, Midwestern University, Glendale, Arizona, USA; ^4^A.T. Still University School of Osteopathic Medicine in Arizona, Mesa, Arizona, USA

**Keywords:** normomatremia, osmotic demyelination syndrome, sodium, traumatic brain injury

## Abstract

Osmotic demyelination syndrome (ODS) is a rare complication associated with rapid sodium changes, typically encountered in patients with severe hyponatremia. ODS in patients with normonatremia (ODSIN) is less recognized. We describe a patient with MRI-detected ODSIN following neurotrauma and reviewed the relevant literature. We present a 57-year-old female with subdural hematoma following ground-level fall. Her initial sodium was 140 mEq/L but over 2 days, rose 17 mEq/L, peaking at 157 mEq/L. On exam, unexplainable, unexpected left-sided hemiplegia with weakness sparing her face were noted; ODS was suspected. MRI revealed central pontine T2 hyperintensity, T1 hypointensity, and FLAIR hyperintensity. Treatment included gradual lowering of sodium with normal saline and free water. She was discharged to a skilled nursing facility (SNF) with sodium 138 mEq/L and upon 4-year follow-up had moderate disability and required some assistance to support activities of daily living. Our literature search yielded 23 cases (22 normonatremic; 1 where normonatremia progressed to hypernatremia). Common signs/symptoms were hyperreflexia, dysarthria, and gait disturbance. Common comorbidities were alcoholism, dialysis, and renal disease/failure. Cranial MRI confirmed all cases, frequently revealing central pontine T2 and FLAIR hyperintensity and T1 hypointensity. Our review further characterizes the diverse etiologies, clinical course, and radiographic features of ODSIN. Clinicians should consider this diagnosis when neurological symptoms occur even in the setting of normonatremia.

## 1. Introduction

Osmotic demyelination syndrome (ODS) is a rare but devastating complication typically associated with rapid correction of low sodium levels. ODS commonly affects the central pons but can extend to extra–pontine regions, including the midbrain, corona radiata, basal ganglia, and thalamus. The detrimental effect of ODS is the decrease in osmolarity and loss of water, which can lead to glial dehydration, myelin breakdown, and/or oligodendroglial apoptosis [1]. ODS is confirmed using magnetic resonance imaging (MRI) with FLAIR sequence. ODS has been found to be associated with chronic alcoholism, malnutrition, and various electrolyte imbalances. While ODS typically occurs in patients with chronic or severe hyponatremia, ODS in normonatremia (ODSIN) is less frequently recognized.

Whereas ODS in severe hyponatremia is typical, it is not often recognized in traumatic brain injury (TBI) patients with normal sodium concentrations. Normal and hypertonic saline are commonly used in this setting to drive sodium concentrations upward (e.g., > 145 mEq/L) to minimize brain swelling. Research has shown benefit with this practice but deleterious sequelae, particularly ODS, have not been well described. We present a patient with ODSIN and provide a systematic review of the available literature to characterize ODSIN's diverse etiologies, clinical course, and radiographic features. No written consent was obtained from the patient as there was no patient-identifiable data included in the report.

## 2. Case Presentation

We report a 57-year-old female with history of hypertension, gastroesophageal reflux disease, alcoholism, and hiatal hernia who presented following ground-level fall. Initially, at an outside hospital, her Glasgow Coma Score (GCS) was 14 with unilateral weakness but rapidly deteriorated to eight [2]. She was intubated, given mannitol, and transferred to our institution. Upon arrival, computed tomography (CT) scan revealed a left-sided subdural hematoma with 16 mm midline shift. Craniotomy was performed to evacuate the hematoma. The operation was successful, characterized by an improvement in shift and brain expansion. Postoperatively, the patient opened her eyes to loud voice and followed simple commands with GCS of 10T.

**Figure 1:**
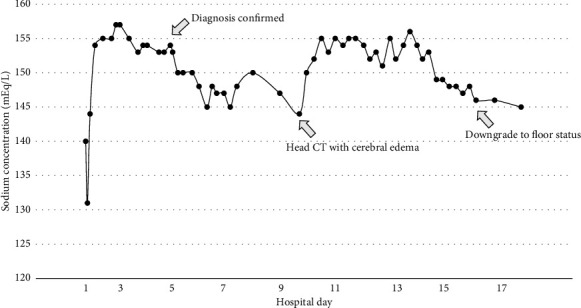
Sodium values during intensive care unit admission.

**Figure 2:**
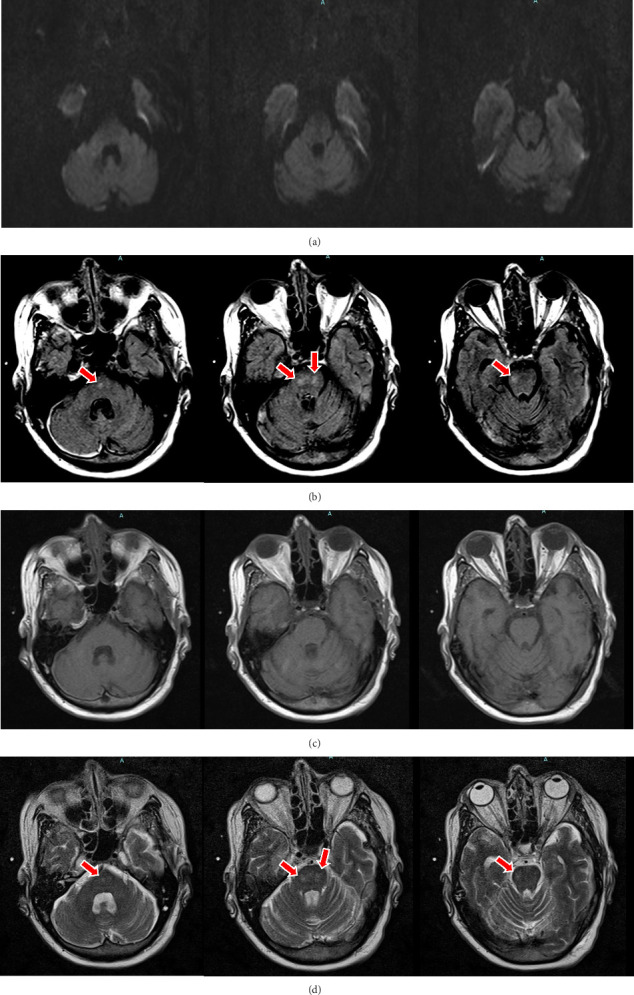
Magnetic resonance imaging brain. (a) The DWI images of the pons appear to be within the normal limit. (b) FLAIR images reveal patchy hyperintensities within the pons. (c) The T1 images appear to be within the normal limit. (d) The T2 images again reveal a hyperintensity within the pons.

The initial sodium (preoperative) was 140 mEq/L. Over 24 h, her sodium rose 17 mEq/L and peaked at 157 mEq/L (Figure 1). Her GCS remained 10T, but she developed an unexpected, unexplainable left-sided hemiplegia with left-sided arm and leg weakness sparing her face. Her sodium over those 24 h ranged between 153 and157 mEq/L. Her cervical spine MRI showed moderate degenerative disc disease (DGD) C5 to C6 with uncovertebral joint hypertrophy, but no spinal cord injury. The MRI showed no restricted diffusion or stroke. There was no organic explanation for her left-sided weakness. An MRI of the brain revealed central pontine T2 hyperintensity, T1 hypointensity, and FLAIR hyperintensity, and ODS was suspected (Figure 2).

Treatment comprised of gradually lowering sodium with normal saline and free water flushes. Over 4 days, her GCS ranged between 11 and 15. On hospital Day 9, she became extremely lethargic and GCS fell to 10 (E3, V2, and M5). A stat CT scan revealed significant cerebral edema and hydrocephalus but stable SDH. EEG was negative for seizures, but prophylaxis was changed from levetiracetam to lacosamide. Free water flushes were discontinued, and 2% sodium chloride was administered to maintain sodium goal above 145 mEq/L. The following day, her neurologic exam improved (GCS, 14) and 2% sodium chloride was discontinued. Over the next few days, her neurologic function remained stable, free water flushes resumed, and on hospital Day 16, she was downgraded to floor status with sodium of 146 mEq/L. She was discharged to skilled nursing facility (SNF) on hospital day 30 with sodium of 138 mEq/L and GCS of 15. Follow-up at 4 years indicated moderate disability, and she requires some assistance to support activities of daily living. She scored 60 on the Barthel Index (Table 1a), 7 on the Lawton–Brody Instrumental Activities of Daily Living (I.A.D.L.) Scale (Table 1b), and three on the Modified Rankin Scale (mRS) (Table 1c) [3–5].

## 3. Discussion

Trauma is one of the leading causes of mortality globally with an annual occurrence of over six million deaths per year [6]. TBI is a major cause of death in trauma patients, with close to 33% of TBI patients dying in the hospital and 33% having poor neurological recovery [7]. Management strategies for severe TBI focus on preventing secondary brain injury by preventing cerebral ischemia and optimizing cerebral perfusion. Hypertonic saline is widely used to increase cerebral perfusion by reducing intracranial pressure and cerebral edema, which is the main cause of death associated with TBI [7, 8]. Many studies have suggested that continuous infusion of hypertonic therapy can decrease the likelihood of intracranial hypertension and increase the probability of survival in the setting of TBI [7, 9, 10]. In a systematic review and meta-analysis of seven clinical trials (*n* = 1521) with patients who received continuous hypertonic saline for brain injury, the odds ratio for in-hospital mortality was 0.74 (95% CI 0.57–0.95) in the treated group [9].

Though hyperosmolar therapy is beneficial to TBI, one pitfall is the risk of ODS. Studies have demonstrated ODS occurring after rapid correction of hyponatremia [11, 12], hyperosmolar hyperglycemic state (HHS) [13–17], hyperammonemia [18, 19] and continuous hyperbilirubinemia [20], hypernatremia [21–27], hypokalemia [28, 29], hyperglycemia [30, 31], and other electrolytic imbalances [32, 33]. We report the first known case of ODSIN without other electrolyte imbalances following TBI. This patient was distinct from other reviewed cases due to deviation in the sodium level from normal to high. She experienced subdural hematoma with shift, unlike other ODSIN cases which did not involve neurotrauma. She experienced common symptoms of ODS including coma, respiratory distress/failure, hemiparesis, lethargy, and dysarthria, and common comorbidities including hypertension and alcoholism. She had typical MRI findings in the central pons including T2 hyperintensity, T1 hypointensity, and FLAIR hyperintensity. Unlike most other cases, she received hypertonic saline and now has moderate disability and requires some support.

## 4. Literature Review

A literature search was performed using PubMed from 1960 to present with the following index terms, “(osmotic demyelination) NOT (hyponatremia),” “(osmotic demyelination) AND (normonatremia),” “(osmotic demyelination) AND (normonatremic),” “(osmotic demyelination) AND (eunatremia),” and “(osmotic demyelination) AND (eunatremic).” Any papers that provided no clinical, radiographic, or pathological evidence of ODSIN were excluded. We excluded any papers with electrolyte imbalances, including hyponatremia, hypernatremia, hypokalemia, hypophosphatemia, and hyperglycemic hyperosmolar state, and non-English papers. References of retrieved manuscripts were reviewed to identify additional cases. Each paper was read by one of three authors, and a second author reviewed papers to ensure standardized and complete data abstraction. Information regarding age, sex, signs and symptoms, comorbidities, brain location, radiographic and pathological findings, clinical data, treatments, and outcomes were abstracted. Not all data were available uniformly due to absence of complete data on each patient due to the study's retrospective nature. We indicate the number of published cases that include relevant information for each characteristic.

## 5. Literature Review Results

Our literature search yielded 258 articles. After inclusion/exclusion criteria screening, there were 23 ODSIN cases reported, none of which were patients with TBI. We excluded cases with hypernatremia at onset or electrolyte imbalances, including hyponatremia, hypokalemia, hypophosphatemia, hyperglycemic hyperosmolar state, hyperammonemia, hyperglycemia, and others. We excluded any papers that provided no clinical, radiographic, or pathological evidence of ODSIN and papers that were in non-English language.

One case report described ODS in a patient with normonatremia (initial sodium, 140 mEq/L) that progressed to hypernatremia (152 mEq/L) while the remaining 22 occurred in normonatremic patients who did not develop hypernatremia. Tables 2, 3, 4a, 4b, 4c, 4d, 4e, 4f, 5, 6, and 7 represent results from the literature search in addition to the case patient.

### 5.1. Normonatremic to Hypernatremic ODS

This case described a 30-year-old male with primary sclerosing cholangitis [34]. He was awaiting liver transplant and became unresponsive on admission Day 65. His exam showed jaundice, extensor posturing to central pain, and absent brainstem reflexes other than intermittent cough reflex (Table 2). Labs showed elevated bilirubin (955 *μ*mol/L), which had fluctuated between 591 and 994 *μ*mol/L throughout his admission (Table 3). Eleven days prior, his sodium had increased from 140 to 152 mmol/L over 4 days. Ammonia and other electrolytes were normal. CT revealed hypoattenuation of internal capsules, thalami, midbrain, and pons (Table 4a). MRI revealed pontine T2 hyperintensity and T1 hypointensity and several extrapontine structures, which were diffusion-restricted and gadolinium-enhanced along peripheral margins (Table 4b). These findings were consistent with ODS. While this case describes ODS in a setting where the sodium level began within a normal range and subsequently increased to a hypernatremic value (similar to what was described in our case report), this patient demyelinated in the setting of liver failure, as compared with our patient who had incurred TBI.

### 5.2. Normonatremic ODS

There were 22 patients (10 males and 12 females), with mean age of 44.9 ± 15.7 years [1, 35–54]. Signs and symptoms are shown in Table 2. All patients had signal abnormalities in the central pons. The most common MRI findings within central pons included T2 hyperintensity (19 patients), T1 hypointensity (14 patients), and FLAIR hyperintensity (nine patients). The second-most commonly affected location was the corona radiata, showing similar findings to central pons. The full list of affected locations and findings can be found in Table 4b. All patients were diagnosed using MRI. Nine patients in this group underwent CT (*n* = 9). Findings on CT included pontine hypodensity (five patients), normal results (four patients), and midbrain hypodensity (two patients). All objective findings can be found in Table 4.

Twelve cases reported specific sodium values (mean initial Na^+^: 137 ± 2.6). The most common signs and symptoms were hyperreflexia (50%), dysarthria (45%), gait disturbance (36%), and dysphagia (32%) (Table 2). The most common comorbidities were alcoholism (41%), dialysis (27%), renal disease/failure (27%), malnutrition (18%), liver failure (18%), and pneumonia (18%) (Table 5). Eleven cases in this group reported laboratory findings (*n* = 11). The most common included elevated BUN/creatinine (55%), anemia (36%), and elevated hepatic transaminases (18%) (Table 3). Neurophysiological findings were reported in three patients and summarized in Table 6. There was substantial treatment variability noted which is highlighted in Table 7. A prognosis was generally good; the majority of patients in this group achieved complete recovery (*n* = 11), but two had significant disability, two were ambulant with support, one had incomplete recovery, and three died.

The two case groups describing ODSIN in the literature were normonatremic patients (*n* = 22) and those with normonatremia that progressed to hypernatremia (*n* = 1). Our case patient falls into the latter category. Due to the very rare nature of the second category, it is challenging to make distinctions between the pathogenesis of ODSIN between the two groups. However, the most common signs and symptoms in the normonatremia group were hyperreflexia (11 patients), dysarthria (10 patients), gait disturbance (eight patients), and dysphagia (seven patients), compared with coma (two patients) in the normonatremia to hypernatremia group. Common comorbidities in the normonatremia group included alcoholism (nine patients), dialysis (six patients), and renal disease/failure (six patients) compared with alcoholism (one patient) and hypertension (one patient) in the normonatremia to hypernatremia group. Both groups showed similar MRI findings. In the normonatremia group, T2 hyperintensity (19 patients), T1 hypointensity (14 patients), FLAIR hyperintensity (nine patients), and restricted diffusion (seven patients) all occurred within the central pons. Similarly, the normonatremia to hypernatremia group experienced T2 hyperintensity (two patients), T1 hypointensity (two patients), and FLAIR hyperintensity (two patients) also within the central pons. There was high treatment variability across both groups, but majority of patients in the normonatremia group received no treatment relevant to their ODS (*n* = 13). In the second group, the two treatment modalities used were high-dose methylprednisolone (one patient) and hypertonic saline (one patient).

Our case patient is a 57-year-old female who suffered a left-sided subdural hematoma following ground-level fall. She had a history of hypertension, GERD, alcoholism, and hiatal hernia and presented with dysarthria, coma, respiratory failure, hemiparesis, lethargy, anemia, and normal initial sodium of 140 mEq/L which later fell to 130 mEq/L. She underwent craniotomy for hematoma evacuation, and subsequently, her sodium increased to 157 mEq/L. An unexplainable left-sided hemiparesis was found on exam, and ODS was suspected. MRI showed central pontine T2 hyperintensity, T1 hypointensity, and FLAIR hyperintensity, findings associated with ODS. Treatment included gradual lowering of sodium with 0.9% sodium chloride and free water. She was discharged to SNF with sodium 138 mEq/L and upon 4-year follow-up, has moderate disability, and requires some support.

Historically, ODS has been associated with long bouts of quickly corrected hyponatremia; however, it is rarely observed in normonatremic patients. This systematic literature review aimed to seek out cases to better describe the prevalence of ODS in normonatremia (ODSIN). To the extent of our awareness, no comprehensive review of ODSIN exists, although few case reports on the rare disease have been published. In this review, we compiled all published case reports that describe ODSIN, which typically occurs in patients with alcoholism, renal disease, liver failure, and pneumonia. The most common findings are T2 hyperintensity, T1 hypointensity, and FLAIR hyperintensity within the central pons on cranial MRI, as well as hyperreflexia and dysarthria. Physicians should keep this in their differential and use caution when correcting sodium values to prevent this rare disease. Being mindful of the type of hyperosmolar agent and level of hyperosmolarity is encouraged when correcting electrolyte imbalances [55].

Due to the retrospective nature of this study, not all data are uniformly available due to the absence of complete data on each patient. Furthermore, the heterogeneity of the cases in this review can pose a challenge, as ODSIN can manifest in various clinical settings. Further research via the prospective collection of clinical data on ODSIN in a database would further delineate the characteristics of this disease more accurately.

In conclusion, ODSIN is a rare complication encountered with the administration of hypertonic IV fluids. This review provides a detailed overview of ODSIN including the first reported episode of ODSIN in the setting of neurotrauma. Given the widespread use and benefit of hypertonic saline following TBI, clinicians should be aware of this potential complication.

## Figures and Tables

**Table 1a:** Long-term (4 years) neurologic status. Barthel Index.

Feeding	10
Bathing	5
Grooming	5
Dressing	10
Bowels	0
Bladder	0
Toilet use	0
Transfers	10
Mobility	10
Stairs	5
Total (0–100)	60

**Table 1b:** Long-term (4 years) neurologic status. Lawton–Brody instrumental activities of daily living scale.

A. Ability to use telephone	1
B. Shopping	1
C. Food preparation	1
D. Housekeeping	1
E. Laundry	1
F. Mode of transportation	0
G. Responsibility for own medications	1
H. Ability to handle finances	1
Total score (0–8)	7

**Table 1c:** Long-term (4 years) neurologic status. Modified Rankin Scale.

No symptoms	0
No significant disability. Able to carry out all usual activities, despite some symptoms	1
Slight disability. Able to look after own affairs without assistance but unable to carry out all previous activities	2
Moderate disability. Requires some help but able to walk unassisted	3
Moderately severe disability. Unable to attend to own bodily needs without assistance and unable to walk unassisted	4
Severe disability. Requires constant nursing care and attention, bedridden, and incontinent	5
Dead	6

**Table 2:** Signs and symptoms.

Signs and symptoms	Normal (*n* = 22), *n* (%)	Normal to high (*n* = 2), *n* (%)
Hyperreflexia	11 (50)	
Dysarthria	10 (45)	1 (50)
Gait disturbance	8 (36)	
Dysphagia	7 (32)	
Lethargy	6 (27)	1 (50)
Nausea	6 (27)	
Quadriparesis/quadriplegia	6 (27)	
Lateral gaze paresis	5 (23)	
Vomiting	5 (23)	
Ataxia of trunk/limbs	5 (23)	
Coma	3 (14)	2 (100)
Respiratory distress/failure	4 (18)	1 (50)
Mild hemiparesis	4 (18)	1 (50)
Hemodynamic instability	4 (18)	
Binocular diplopia	3 (14)	
Anorexia	3 (14)	
Syncope	3 (14)	
Gaze-evoked nystagmus	3 (14)	
Tonic–clonic convulsions/seizures	3 (14)	
Spasticity	3 (14)	
Asymptomatic	3 (14)	
Bifacial palsy	3 (14)	
Neurological deterioration (without detailed description)	2 (9)	
Psychomotor slowing	2 (9)	
Akinetic/rigid/Parkinsonism like	2 (9)	
Babinski sign absent	2 (9)	
Locked-in state	2 (9)	
Vertigo	2 (9)	
Fatigue	2 (9)	
Acute headache	2 (9)	
Bilateral facial hypoesthesia	2 (9)	
Decreased palatal movements	2 (9)	
Resting and positional tremor	1 (5)	
Behavioral changes	1 (5)	
Diffusely reduced deep tendon reflexes	1 (5)	
Hallucinations	1 (5)	
Diminished gag reflex	1 (5)	
Apathetic	1 (5)	
Emotional incontinence	1 (5)	
Suicidal ideation	1 (5)	
Short-term memory impairment	1 (5)	

**Table 3:** Laboratory findings.

	Normal (*n* = 11), *n* (%)	Normal to high (*n* = 2), *n* (%)
Elevated BUN/creatinine	6 (55)	
Anemia	4 (36)	1 (50)
Elevated hepatic transaminases	2 (18)	
Elevated bilirubin	1 (9)	1 (50)
Leukocytosis	1 (9)	
Low prealbumin	1 (9)	
Low serum albumin	1 (9)	
Low CD4+	1 (9)	
Mild hypokalemia	1 (9)	
Low platelet count	1 (9)	
Slight hyperglycemia	1 (9)	
Elevated cholesterol	1 (9)	
Elevated uric acid	1 (9)	
Elevated PTH	1 (9)	

**Table 4a:** Objective findings. Computed tomography brain findings.

	Normonatremia (*n* = 9), *n* (%)	Normal-- > high (*n* = 1), *n* (%)
Pons		
Hypodensity	5 (56)	1 (100)
Normal	4 (45)	
Midbrain		
Hypodensity	2 (22)	1 (100)
Mesencephalon		
Hypodensity	1 (11)	
Internal capsules		
Hypodensity		1 (100)
Thalami		
Hypodensity		1 (100)

**Table 4b:** Objective findings. Magnetic resonance imaging brain findings.

	Normonatremia (*n* = 22), *n* (%)	Normal-- > high (*n* = 2), *n* (%)
Central pons		
T2 hyperintensity	19 (86)	2 (100)
T1 hypointensity	14 (64)	2 (100)
FLAIR hyperintensity	9 (41)	2 (100)
Restricted diffusion	7 (32)	1 (50)
High signal on ADC	1 (5)	
T1 with gadolinium enhancements present	2 (9)	
T1 without gadolinium enhancements present	1 (5)	
Corona radiata		
T2 hyperintensity	3 (14)	
T1 hypointensity	2 (9)	
FLAIR abnormal signal intensity	2 (9)	
Basal ganglia		
T2 hyperintensity	1 (5)	
FLAIR hyperintensity	1 (5)	
Caudate nucleus		
T2 hyperintensity	1 (5)	
T1 hypointensity	1 (5)	
FLAIR hyperintensity	1 (5)	
Lentiform nucleus		
FLAIR hyperintensity	1 (5)	
Putamen		
T2 hyperintensity	1 (5)	
T1 hypointensity	1 (5)	
FLAIR hyperintensity	1 (5)	
Internal capsule		
T2 hyperintensity	2 (9)	
T1 hypointensity	1 (5)	
Centrum semiovale		
T2 hyperintensity	1 (5)	
T1 hypointensity	1 (5)	
FLAIR hyperintensity	1 (5)	
Diencephalon/thalamus		
T2 hyperintensity	2 (9)	1 (50)
T1 hypointensity	1 (5)	1 (50)
FLAIR hyperintensity	1 (5)	1 (50)
Restricted diffusion	1 (5)	
T1 with gadolinium enhancements present	1 (5)	
Mesencephalon/midbrain		
T2 hyperintensity		1 (50)
FLAIR hyperintensity		1 (50)
T1 hypointensity		1 (50)
Restricted diffusion	1 (5)	
T1 with gadolinium enhancements present	1 (5)	

**Table 4c:** Objective findings. Electroencephalogram findings.

	Normonatremia (*n* = 5), *n* (%)	Normal-- > high (*n* = 1), *n* (%)
Normal	3 (60)	
Diffuse bihemispheric slowing	1 (20)	1 (100)
Nonconvulsive status epilepticus	1 (20)	
Left hemispheric PLEDs		1 (100)
Nonepileptiform sharp waves		1 (100)

**Table 4d:** Objective findings. Cerebrospinal fluid findings.

	Normonatremia (*n* = 7), *n* (%)	Normal-- > high (*n* = 0), *n* (%)
Normal	6 (86)	
Mild lymphocyte pleocytosis	1 (14)	
Elevated protein	1 (14)	

**Table 4e:** Objective findings. Magnetic resonance angiography findings.

	Normonatremia (*n* = 2), *n* (%)	Normal-- > high (*n* = 0), *n* (%)
Normal	2 (100)	

**Table 4f:** Objective findings. Carotid artery sonography findings.

	Normonatremia (*n* = 1), *n* (%)	Normal-- > high (*n* = 0), *n* (%)
Carotid atherosclerosis of right internal carotid with significant hemodynamic change	1 (100)	

**Table 5:** Comorbidities.

	Normal (*n* = 22), *n* (%)	Normal to high (*n* = 2), *n* (%)
*Comorbidities*		
Alcoholism	9 (41)	1 (50)
Dialysis	6 (27)	
Renal disease/failure	6 (27)	
Malnutrition	4 (18)	
Liver failure	4 (18)	
Pneumonia	4 (18)	
Hypertension	3 (14)	1 (50)
Depression	3 (14)	
Peripheral neuropathy	2 (9)	
Septicemia	2 (9)	
HIV	2 (9)	
Hyperlipidemia	2 (9)	
Gastritis	2 (9)	
Anterior circulation stroke	1 (5)	
Miller Fisher variant of Guillain–Barre syndrome	1 (5)	
Brain stem infarction	1 (5)	
Dehydration	1 (5)	
Tuberculosis meningitis	1 (5)	
Chronic secretory diarrhea	1 (5)	
Disseminated aspergillosis	1 (5)	
Toxoplasmosis	1 (5)	
Pseudobulbar palsy	1 (5)	
Carcinoma of cervix	1 (5)	
Thyroid nodules	1 (5)	
Renal angiomyolipoma	1 (5)	
Lumbago	1 (5)	
Anxiety	1 (5)	
Homozygous alpha-thalassemia	1 (5)	
Coronary artery disease	1 (5)	
Benign prostate hyperplasia	1 (5)	
Diffuse large B-cell lymphoma	1 (5)	
Type II diabetes	1 (5)	
Peripheral arterial obstruction	1 (5)	
Hypochondria	1 (5)	
Chronic headache	1 (5)	
Upper GI bleeding	1 (5)	
Hepatic encephalopathy	1 (5)	
Bacterial peritonitis	1 (5)	
Abdominal trauma	1 (5)	
Pelvic fracture	1 (5)	
Gastroesophageal reflux disease		1 (50)
Hiatal hernia		1 (50)
Traumatic brain injury		1 (50)
Acute subdural hematoma		1 (50)
Epilepsy		1 (50)
Hematinics	1 (5)	

**Table 6:** Neurophysiology.

	Normonatremia (*n* = 3), *n* (%)	Normal-- > high (*n* = 0), *n* (%)
Brainstem auditory–evoked potentials		
Abnormal prolongations in the I–VI interpeak latencies	1 (33)	
Auditory brainstem responses		
Bilateral sensory neural hearing loss—greater on one side	1 (33)	
Electromyography/nerve conduction studies		
Hypoglossal denervation	1 (33)	
Sensory neuropathy	1 (33)	
Somatosensory-evoked potentials		
Decreased N20 amplitudes	1 (33)	

**Table 7:** Treatments^∗^ and outcomes.

	Complete recovery, *n* (%)	Significant disability, *n* (%)	Ambulant with support, *n* (%)	Incomplete recovery, *n* (%)	Death, *n* (%)	Total, *n* (%)
Normonatremia (*n* = 19)	11 (58)	2 (11)	2 (11)	1 (5)	3 (16)	19 (100)
Levodopa	1 (5)					1 (5)
IV immunoglobulin			1 (5)			1 (5)
Vitamin supplements				1 (5)		1 (5)
K+ replacement + methylprednisolone pulse therapy + prednisolone	1 (5)					1 (5)
Methylprednisolone	1 (5)					1 (5)
Ringer's lactate + 5% albumin	1 (5)					1 (5)
No treatment	7 (37)	2 (11)	1 (5)		3 (16)	13 (68)
Normal to high (*n* = 2)	0	0	1 (50)	0	1 (50)	2 (100)
Methylprednisolone					1 (50)	1 (50)
Hypertonic saline			1 (50)			1 (50)
Total						21

∗Table represents treatments relevant to ODS.

## Data Availability

Data sharing is not applicable to this article as no new data were created or analyzed in this study.
